# Transcriptomic changes throughout post-hatch development in *Gallus gallus* pituitary

**DOI:** 10.1530/JME-16-0186

**Published:** 2016-12-03

**Authors:** Elizabeth M Pritchett, Susan J Lamont, Carl J Schmidt

**Affiliations:** 1 Animal and Food ScienceUniversity of Delaware, Newark, Delaware, USA; 2 Animal ScienceIowa State University, Ames, Iowa, USA

**Keywords:** chicken, pituitary, gene expression, development

## Abstract

The pituitary gland is a neuroendocrine organ that works closely with the hypothalamus to affect multiple processes within the body including the stress response, metabolism, growth and immune function. Relative tissue expression (rEx) is a transcriptome analysis method that compares the genes expressed in a particular tissue to the genes expressed in all other tissues with available data. Using rEx, the aim of this study was to identify genes that are uniquely or more abundantly expressed in the pituitary when compared to all other collected chicken tissues. We applied rEx to define genes enriched in the chicken pituitaries at days 21, 22 and 42 post-hatch. rEx analysis identified 25 genes shared between all time points, 295 genes shared between days 21 and 22 and 407 genes unique to day 42. The 25 genes shared by all time points are involved in morphogenesis and general nervous tissue development. The 295 shared genes between days 21 and 22 are involved in neurogenesis and nervous system development and differentiation. The 407 unique day 42 genes are involved in pituitary development, endocrine system development and other hormonally related gene ontology terms. Overall, rEx analysis indicates a focus on nervous system/tissue development at days 21 and 22. By day 42, in addition to nervous tissue development, there is expression of genes involved in the endocrine system, possibly for maturation and preparation for reproduction. This study defines the transcriptome of the chicken pituitary gland and aids in understanding the expressed genes critical to its function and maturation.

## Introduction

The avian pituitary gland is a neuroendocrine organ that works closely with the hypothalamus to regulate multiple processes within the body including the stress response, reproduction, metabolism, growth and immune function. The hypothalamus receives neuronal and endocrine signals from the brain and body and synthesizes releasing hormones that are sent to the pituitary gland to either inhibit or stimulate the production of several hormones that will directly affect other tissues (i.e. growth hormone (GH)) or stimulate the release of further protein products (i.e. adrenocorticotropic hormone (ACTH)). The posterior pituitary gland, or the neurohypophysis, is composed of nervous tissue and is connected to the hypothalamus via the median eminence. Neurosecretory terminals within the posterior pituitary store and release arginine, vasotocin (AVT) and mesotocin that are synthesized in the supraoptic nucleus and paraventricular nucleus of the hypothalamus, respectively. Releasing hormones synthesized by the hypothalamus are transported to the anterior pituitary gland through the hypophyseal blood portal system and regulate the production and release of various endocrine hormones (ACTH, GH, LH, FSH and TSH). Relative tissue expression analysis (rEx) was used to compare the chicken pituitary gland transcriptome to the transcriptome of all other tissues our laboratory has analyzed in the broiler chicken across three time points: days 21, 22 and 42 post-hatch. Previous work focusing on the morphometric changes of broiler chickens post-hatch identified two distinct growth periods: one from day of hatch through day 14, and one from day 14 to day 42 or typical market age (Schmidt *et al*. 2009). The days discussed fall into the second growth phase. These days were also chosen as part of a larger heat stress study conducted in our laboratory. The pituitary gland is a complex tissue in that it contains many different cell types, and signals are sent through neural endocrine, paracrine and autocrine networks. The messages received by the pituitary gland lead to many changes within the tissue and results in tissue plasticity. The results of this analysis identify the expression levels of genes in the pituitary gland that are either more abundant or are unique to this particular tissue at specific points in development. These data confer a better understanding of the genes and pathways that are critical to the function of the pituitary gland.

## Materials and methods

### Animal housing and tissue collection

Male Ross 708 broiler chickens (*Gallus gallus*) were obtained on the day of hatch from Mountaire Hatchery (Millsboro, DE, USA) and placed in large-colony houses on the University of Delaware farm (Newark, DE, USA). Husbandry and management followed all standards and procedures as approved by the Animal Care and Use Committee (AACUC #(27) 03-12-14R). Standard broiler feed (corn-soy), which met all NRC requirements (National Research Council 1994), and water were supplied, and animals had *ad libitum* access throughout the trial. Birds were humanely killed and the pituitaries were collected on 21, 22 and 42 days post-hatch as part of a larger study. Whole pituitaries were placed into liquid nitrogen and stored at −80°C until further analysis.

### RNA isolation, cDNA synthesis, Rnaseq library preparation and qRT-PCR validation

Total RNA was extracted from whole pituitary glands (8–20 mg depending on age) from individual birds using the Qiagen RNeasy Mini Kit (Germantown, MD, USA). Total RNA quantity was measured using a Qubit Fluorometer, and quality was assessed by fragment analysis. A total of 15 pituitary glands from day 21, 10 from day 22 and 10 from day 42 were used for individual Rnaseq library preparation using the Illumina Stranded RNAseq kit (San Diego, CA, USA). All 35 samples were sequenced at the Delaware Biotechnology Institute (DBI) Sequencing and Genotyping center using the Illumina HiSeq 2500 sequencer. All libraries were sequenced to a depth of 20–30 million reads per library. The reads were aligned to the *Gallus gallus*, ver 4 genome sequence, identified and counted using software packages Bowtie, Tophat and Cufflinks, and expression levels were reported as fragments per kilobase of gene per million mapped reads (FPKM) values for further analysis (Trapnell *et al*. 2009, Langmead & Salzberg 2012). Three biological replicates for each day (day 21, 22 and 42) were used for cDNA synthesis using the SuperScript First-Strand Synthesis System for RT-PCR (Invitrogen). cDNA concentration was determined using the Qubit Fluorometer and diluted to 30 ng/µL for PCR. qRT-PCR was performed using Fast SYBR green master mix (Applied Biosystems) on the Applied Biosystems 7500 Fast Real-Time PCR system for the following genes: glycoprotein alpha subunit (*CGA*), follicle-stimulating hormone beta (*FSHB*), growth hormone (*GH*), proopiomelanocortin (*POMC*), prolactin (*PRL*) and thyroid-stimulating hormone beta (*TSHB*). Each qRT-PCR (Table 1) was performed in triplicate, and analysis was completed using the delta Ct method. All sequencing data generated in the current project have been uploaded in the NCBI Gene Expression Omnibus application and are accessible through GEO series, accession number GSE89297.

**Table 1:** Primer sequences used for qRT-PCR transcriptome validation.

**Gene**	**Forward primer**	**Reverse primer**
*CGA*	5′ GCAACGTGCTGTGTAGCAAAG 3′	5′ TGGTTCTCTATCTTCACATTGCCTT 3′
*FSHB*	5′ CGTACAGGGTAGAGCCAACGA 3′	5′ GGCCCTCAAAAGGCTGAAC 3′
*GH*	5′ GCTTCAAGAAGGATCTGCACAA 3′	5′ GCGCCGGCACTTCATC 3′
*POMC*	5′ GCTACGGCGGCTTCATGA 3′	5′ CGATGACGTTTTTGAACAGA 3′
*PRL*	5′ TTGGGCGGGTTCATTCTG 3′	5′ GGCCGTCCCAGTGAGAGTAA 3′
*TSHB*	5′ TGGCCATCAACACCACCAT 3′	5′ CGTTGCTGTCCCGTGTCA 3′

Primer sequences used in qRT-PCR validation of transcriptomic data comparing expression levels between days 21, 22 and 42. Alpha subunit of glycoproteins (*CGA*), follicle-stimulating hormone, beta (*FSHB*), growth hormone (*GH*), proopiomelanocortin (*POMC*), prolactin (*PRL*) and thyroid-stimulating hormone, beta (*TSHB*).

### Relative tissue expression analysis

The gene expression of the pituitary gland was compared to the expression of other tissues (abdominal fat pad, heart fat pad, breast muscle, cerebellum, heart, liver, duodenum, jejunum, ileum, spleen, retina, pineal and hypothalamus) analyzed by our laboratory following the relative tissue expression (rEx) protocol outlined by Bailey and coworkers (Bailey *et al*. 2009). Each day the expression was analyzed independently, and maximum FPKM values for each gene in the pituitary gland were compared to the median FPKM value of each gene in all other collected tissues with the following calculation:

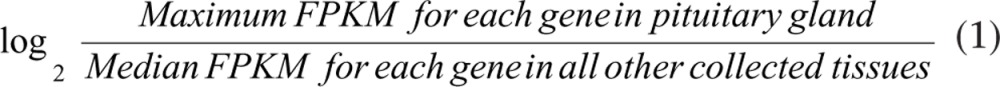


The log_2_ distribution was used to normalize the data and apply a *t*-test. Resulting genes that were greater than two standard deviations from the mean and with a *P* value less than 0.05 were considered enriched in the pituitary gland. The enriched expression of these particular genes is thought to provide the unique functions of the pituitary gland or is critical to the overall function of the pituitary gland at that point in development. The enriched genes were uploaded to AmiGO 2 for gene ontology (GO) term analysis (Carbon *et al*. 2009), PathRings for pathway analysis (Zhu *et al*. 2015) and WebGIVI for text mining and the identification of iTerms (L Sun & CJ Schmidt, unpublished observations).

## Results

**Figure 1:**
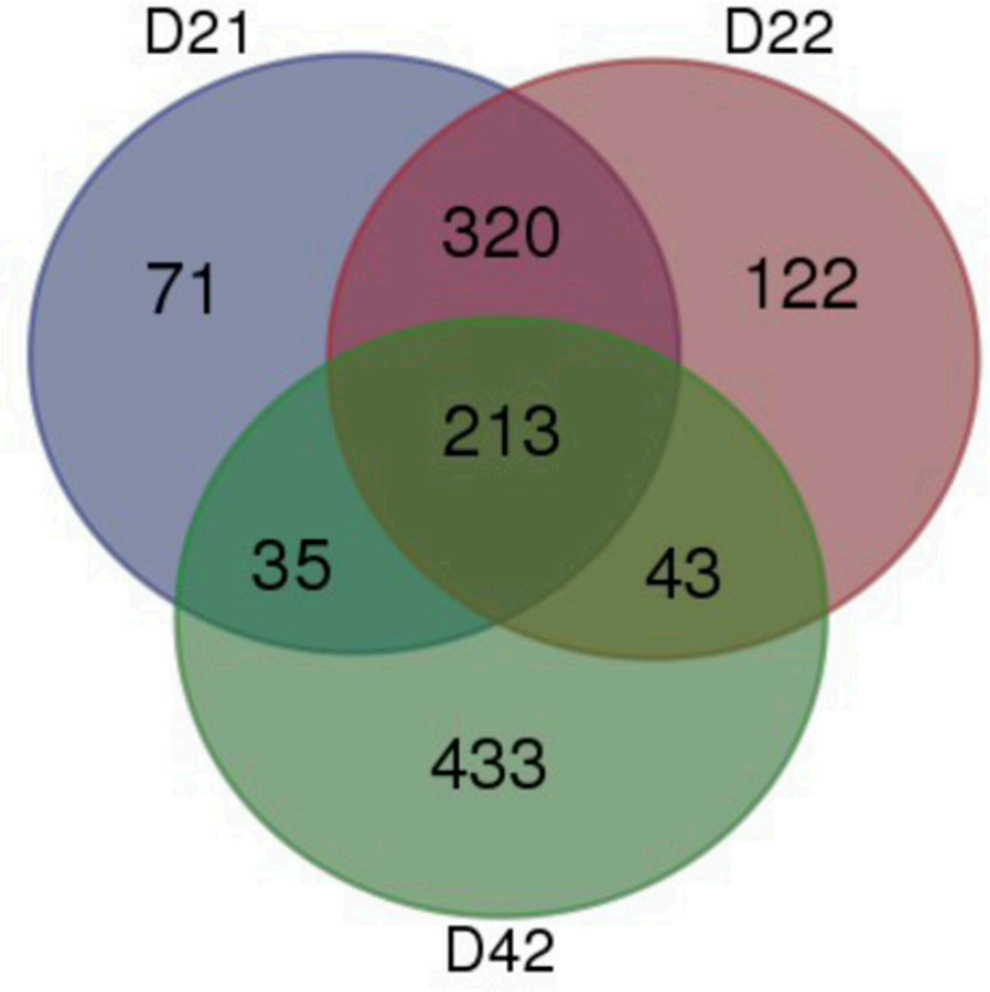
Distribution of enriched genes in the chicken (*Gallus gallus*) pituitary gland by day. Total number of enriched genes after relative tissue expression analysis in the whole pituitary gland at post-hatch days 21, 22, and 42.

Starting gene lists and final enriched genes by day are available in Supplementary Tables 1, 2, 3, 4, 5 and 6 (see section on supplementary data given at the end of this article) (Supplementary Tables 1, 2 and 3; starting gene list for days 21, 22 and 42, respectively; Supplementary Tables 4, 5 and 6; final enriched gene list for days 21, 22 and 42, respectively). Figure 1 shows a Venn diagram of the number of enriched pituitary genes shared (intersects) and differing (complement) between time points. A total of 25 enriched pituitary genes were shared between all three time points. Day 21 and day 22 were chosen for collection as part of a larger study and share 295 enriched genes compared to all other tissues. By day 42, there are 407 enriched genes not shared with day 21 and 22.

### Day 21 and day 22: 295 shared genes

**Figure 2:**
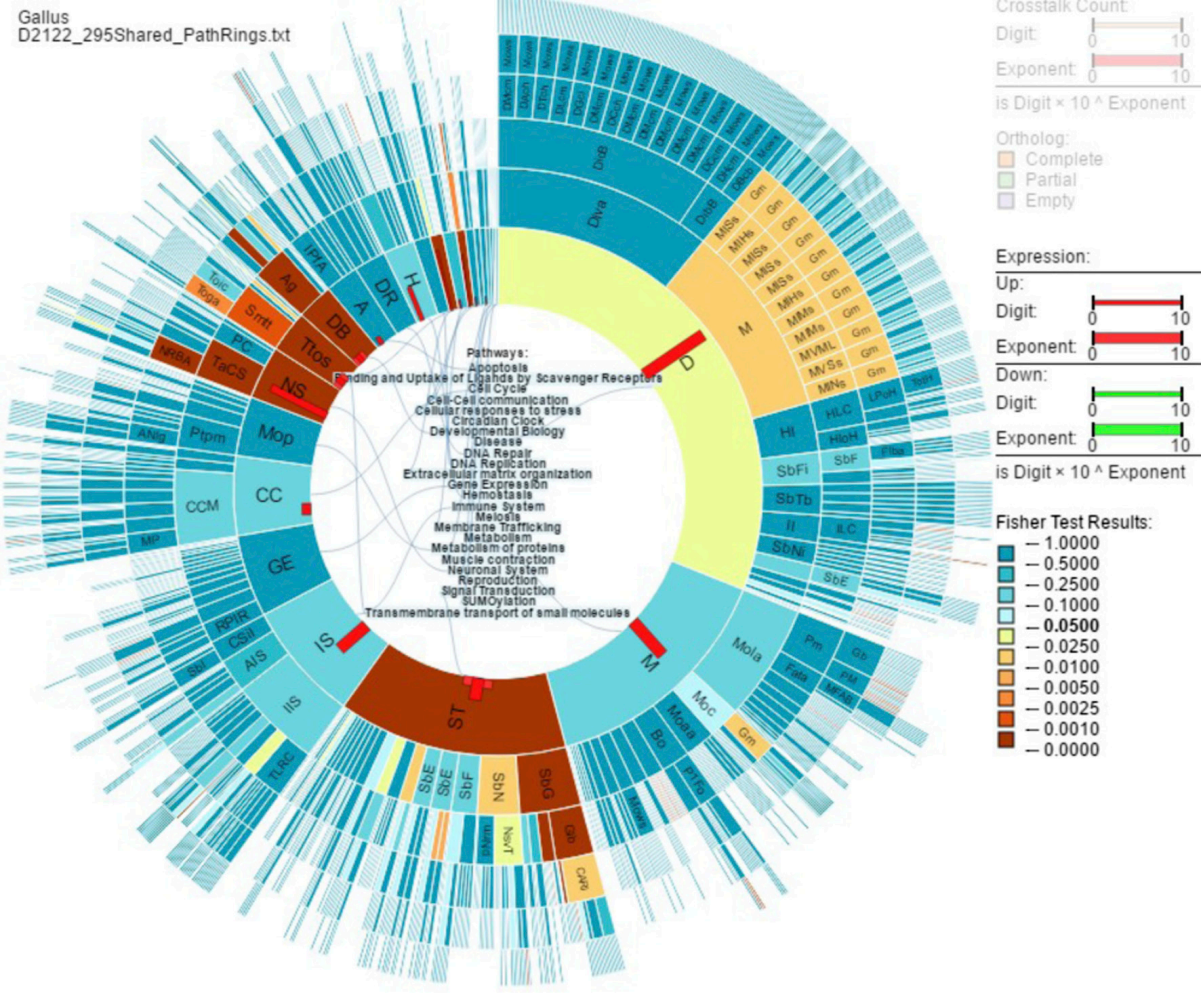
PathRings output for 295 shared enriched genes between days 21 and 22 in chicken (*Gallus gallus*) pituitary glands. Significance, determined by the Fisher Exact Test, is indicated by color. Blue is not significant while yellow to maroon is *P*-value 0.05–0.0001, respectively. DB, developmental biology; NS, neuronal system; ST, significant pathways are signal transduction; Ttos, transmembrane transport of small molecules.

The 295 enriched genes shared between day 21 and day 22 grouped into the following GO terms: structure development (75 genes), neurogenesis (35 genes), neuron differentiation (26 genes) and nervous system development (43 genes). More specific GO terms associated with the 295 shared genes are neuron cell–cell adhesion (4 genes), oligodendrocyte differentiation (8 genes) and regulation of gliogenesis (9 genes). Figure 2 shows PathRings output for 295 shared genes between D21 and D22. Significant pathways and genes are shown in Table 2.

**Table 2:** Significant pathways in PathRings and the enriched genes within each pathway for 295 shared genes between days 21 and 22 in the chicken (*Gallus gallus*) pituitary gland.

**Pathway**	**Gene symbol**
Signal transduction	ADORA1, ARHGEF4, CHRDL1, CNTN1, COL9A3, EGF, ERBB4, FZD10, GFAP, GNAO1, GPR17, GREM2, GRM3, GRM7, GRPR, HRH3, NPBWR1, NRG3, NTRK2, PDE4A, PDE8B, RAMP1, WNT11, WNT6, WNT7B
Neuronal system	CACNG4, CHRNA4, GABRA3, GRIA4, GRIK2, GRIK3, KCNK2
Transmembrane transport of small molecules	ANO4, AQP4, ASIC4, GABRA3, SGK2, SLC13A5, SLC15A2, SLC26A4, SLC30A8, SLC44A5, SLC6A9
Developmental biology	ANK2, CHL1, CNTN1, CNTN2, COL9A3, LAMA1, NFASC, NTN1, SLIT1, UNC5A

DB, developmental biology; NS, neuronal system; ST, signal transduction; Ttos, transmembrane transport of small molecules.

Several genes within the signal transduction pathway and developmental biology pathway were related to axon connection formation and axonal organization (*CNTN1*, *CNTN2*, *NFASC*, *LAMA1* and *SLIT1*), as well as neurite outgrowth (*EGF*, *ERBB4* and *NRG3*). Additionally, glutamate (*GRM3* and *GRM7*) and GABA (*GABRA3*) receptors and genes related to synaptic transmission and messaging (*ASIC4*, *HRH3* and *CHL1*) were enriched at days 21 and 22 when compared to all other tissues.

### Day 21: 74 genes; day 22: 92 genes

Days 21 and 22 share a large portion of enriched genes; however, there are genes unique to each day. Further investigation of the individual days in AmiGO2 indicates an enrichment of genes associated with neuronal morphogenesis and synapse structure and organization at day 21 (*SYNDIG1*, *RELN* and *LRRTM1*). At day 22, the uniquely enriched genes are related to neurite outgrowth, axonal migration, oligodendrocyte differentiation and myelination (*FA2H*, *GLDN*, *PLP1*, *EPHA2*, *PTGDS*, *PTGS1* and *STMN4*). Two immune-related genes were also enriched in day 22: histone deacetylase 11 (*HDAC11*) and complement regulatory protein CD59 molecule (*CD59*). Although these genes were uniquely enriched in the pituitary gland at day 22, the mean FPKM for these genes at days 21 and 22 were both high in comparison to day 42 ((*HDAC11*: day 21: 75.27, day 22: 84.64, day 42: 6.53); (*CD59*: day 21: 467.2, day 22: 505.7, day 42: 54.0)).

### Day 42: 407 genes

**Figure 3:**
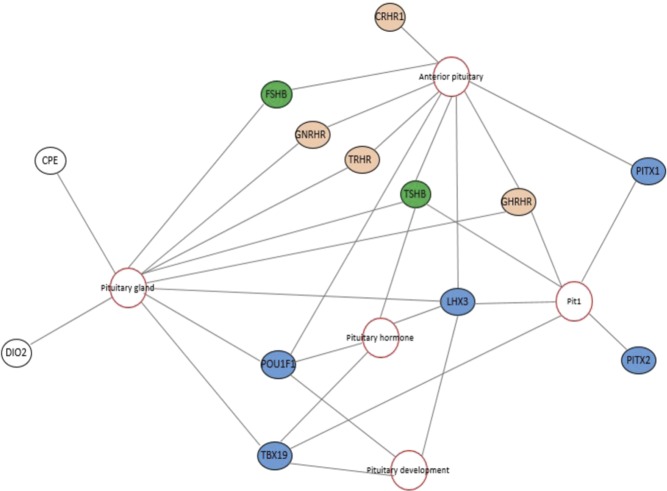
Adapted WebGIVI output for chicken (*Gallus gallus*) pituitary iTerms and enriched genes expressed at day 42. Adapted WebGIVI output for general ‘pituitary’ iTerms. Red circles indicate iTerms generated by WebGIVI. Black outlined circles indicate genes from the enriched gene input list. Gray lines (edges) show connections between genes and iTerms. Blue filled genes are pituitary transcription factors. Orange filled genes are receptors for hypothalamus releasing hormones. Green filled genes are beta subunits of follicle stimulating hormone (FSH) and thyroid stimulating hormone (TSH).

**Figure 4:**
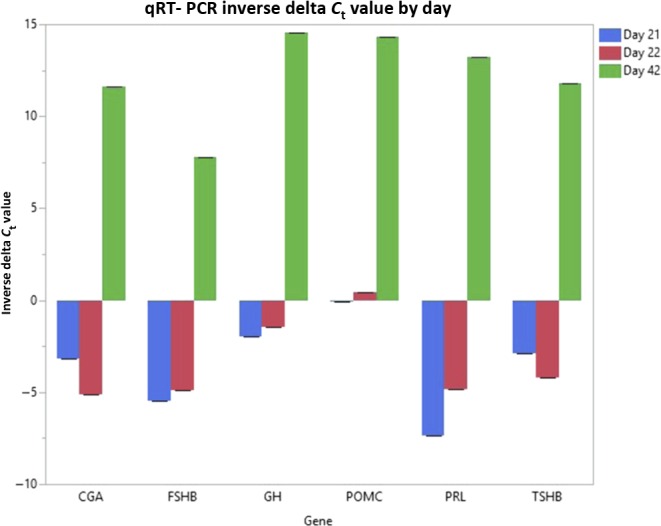
Inverse Delta *C*_t_ values for six genes comparing days 21, 22, and 42 chicken (*Gallus gallus*) post-hatch pituitary gene expression. Alpha subunit of glycoproteins (*CGA*), follicle stimulating hormone, beta (*FSHB*), growth hormone (*GH*), pro-opiomelanocortin (*POMC*), prolactin (*PRL*), and thyroid stimulating hormone, beta (*TSHB*) were used to validate increased expression seen in the transcriptome between days 21, 22, and 42. Inverse Delta *C*_t_ values are shown. Errors bars constructed using one standard error from the mean.

At day 42, there are 407 unique genes with the following GO terms: pituitary gland development (11 genes), endocrine system development (15 genes), hormone metabolic process and regulation of hormone levels (28 genes) and cell–cell signaling (18 genes) (Table 3). In addition to endocrine-related GO terms, neurogenesis (34 genes) and nervous system development (42 genes), GO terms were also present within day 42 enriched genes. Figure 3 shows the pituitary-specific iTerms and genes output from WebGIVI for the day 42 time point. At day 42, expression of endocrine-related genes such as *GH*, *PRL*, *CGA*, *TSHB*, *FSHB* and *POMC* increased and the following fold changes were seen in the transcriptomic data between days 22 and 42: *GH* 2700-fold, *PRL* 3000-fold, *CGA* (alpha subunit for FSHB, TSHB LHB) 1600-fold, *TSHB* 2300-fold, *FSHB* 6800-fold and *POMC* 2600-fold. To validate these robust transcriptomic changes, qRT-PCR results are summarized in Fig. 4.

**Table 3:** Gene ontology (GO) terms and enriched genes within each term for chicken (*Gallus gallus*) day 42 pituitary glands.

**Gene ontology (GO) term**	**# of genes in GO term**	**Gene symbol**
Pituitary gland development	11	DRD2, GHRHR, ISL1, LHX3, PAX6, PITX1, PITX2, POU1F1, SIX3, TBX19, WNT5A
Endocrine system development	16	CGA, CRHR1, DRD2, GHRHR, ISL1, LHX3, NR5A1, PAX6, PITX1, PITX2, POU1F1, RFX6, RFX8, SIX3, TBX19, WNT5A
Regulation of hormone levels	17	CGA, CPE, CRHR1, DIO2, DRD2, ESR1, FOXL2, GHRHR, GHSR, ILDR1, INHBA, NR5A1, PASK, PCSK1, POMC, PRL, RFX6
Cell–cell signaling	24	C2CD4C, CACNA1B, CADPS, CGA, CHRNA3, CPE, CRHR1, DRD2, GABRG1, GHRHR, GHSR, GPR149, HTR1B, INHBA, KLF4, LHX5, LOC100858799, P2RX2, PDYN, PENK, PITX2, POMC, SYT4, WNT5A

### Day 21, day 22 and day 42: 25 shared genes

**Figure 5:**
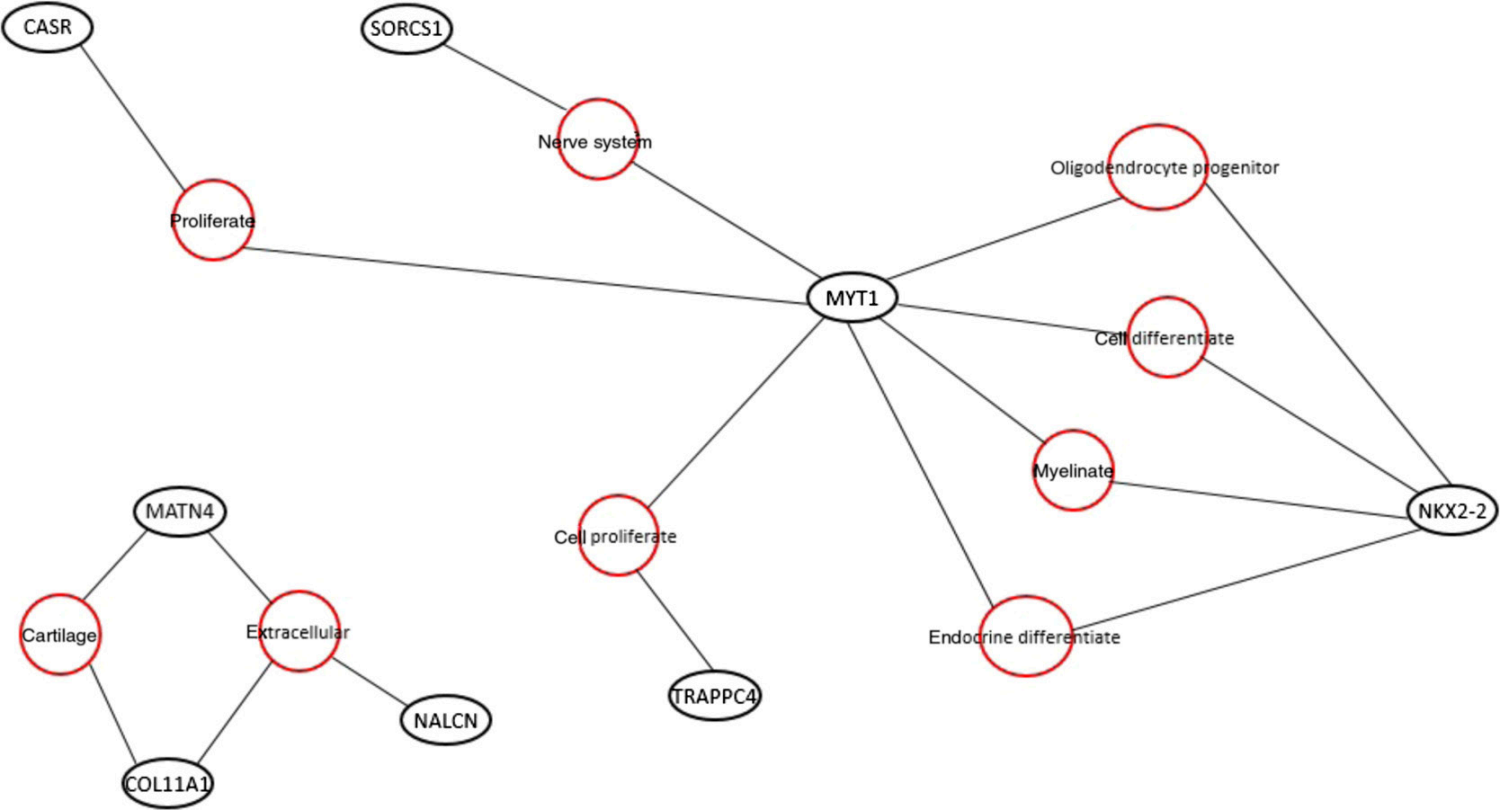
Adapted WebGIVI output for chicken (*Gallus gallus*) pituitary iTerms and 25 shared enriched genes expressed at days 21, 22 and 42. Adapted WebGIVI output for 25 genes enriched at all three time points and corresponding iTerms. Red circles indicate iTerms generated by WebGIVI. Black outlined circles indicate genes from the enriched gene input list. Gray lines (edges) show connections between genes and iTerms.

The 25 enriched genes mean FPKM values for each day are shown in Table 4. Enriched genes were uploaded to WebGIVI (Fig. 5) and AmiGO2 GO terms system development (*COL11A1*, *DNAH5*, *GPC3*, *SIX6*, *SOX3* and *TRAPPC4*) and sensory organ development (*COL11A1*, *SIX6* and *SOX3*). These genes include transcription factors and genes whose products code for structural proteins and are associated with the extracellular matrix.

**Table 4:** Mean FPKM values for 25 shared genes between days 21, 22 and 42 in the chicken (*Gallus gallus*) pituitary gland.

**Gene**	**Description**	**Day 21**	**Day 22**	**Day 42**
ADIRF	Adipogenesis regulatory factor	2.16	1.722	1.8
CASR	Calcium-sensing receptor	2.33	2.57	2.41
COL11A1	Collagen, type XI, alpha 1	17.17	20.74	24.49
DNAH5	Dynein, axonemal, heavy chain 5	0.55	0.4	0.98
FAM135B	Family with sequence similarity 135, member B	1.82	1.82	2.08
FAM3B	Family with sequence similarity 3, member B	5	3.98	3.4
GPC3	Glypican 3	15.17	15.82	12.37
LOC101749214	Uncharacterized LOC101749214	1.68	1.45	0.99
LOC101749680	Uncharacterized LOC101749680	0.53	0.76	1.21
LOC101749716	Uncharacterized LOC101749716	0.95	0.67	1.79
LOC101749894	Homeobox protein Nkx-2.2-like	17.42	20.4	16.02
LOC101750727	Uncharacterized LOC101750727	1777.2	1074.13	958.83
LOC421690	Vacuolar protein 8-like	0.56	0.82	0.61
MATN4	Matrilin 4	2.2	2.1	7.02
MDGA2	MAM domain containing glycosylphosphatidylinositol anchor 2	14.16	16.98	14.25
MOV10L1	Mov10l1, Moloney leukemia virus 10-like 1, homolog (mouse)	4.53	3.53	6.09
MYT1	Myelin transcription factor 1	8.5	8.9	7.35
NALCN	Sodium leak channel, non-selective	14	13.27	10.73
NKX2-2	NK2 homeobox 2	20.34	25.9	18.47
RNF182	Ring finger protein 182	1.43	1.53	3.14
SIX6	SIX homeobox 6	53.92	51.5	44.8
SORCS1	Sortilin-related VPS10 domain containing receptor 1	13.6	14.65	16.97
SOX3	SRY (sex determining region Y)-box 3	4.99	5.04	3.95
TRAPPC4	Trafficking protein particle complex 4	36.72	38.12	34.17
UMODL1	Uromodulin-like 1	1.3	1.52	2.55

Mean FPKM values in the pituitary gland for 25 shared enriched genes in days 21, 22 and 42 when compared to all other tissues (abdominal and heart fat, breast muscle, cerebellum, heart, liver, duodenum, jejunum, ileum, spleen, retina, pineal and hypothalamus).

### Transcription factors

Table 5 shows the transcription factors (TF) enriched by day. In the 295 expressed genes shared between days 21 and 22, there are 14 enriched transcription factors. AmiGO 2 analysis resulted in GO terms negative regulation of neurogenesis and negative regulation of nervous system development (*NTN1*, *PHOX2B*, *SOX8* and *VAX1*). Thirty transcription factors were enriched at day 42 when compared to all other tissues. AmiGO2 analysis resulted in two notable GO terms: pituitary gland development (*LHX3*, *TBX19*, *POU1F1*, *PAX6*, *ISL1*, *PITX1*, *PITX2* and *SIX3*) and endocrine system development (8 genes listed previously and NR5A1 and RFX6). Two transcription factors were present in all three days studied: Myelin Transcription Factor 1 (*MYT1*) and NK2 Homeobox 2 (*NKX2-2*). *MYT1* is a DNA-binding protein with an expression pattern that indicates a potential role in regulating oligodendrocyte differentiation. In the developing and adult central nervous system, *MYT1* expression correlates with oligodendrocyte cell growth (Nielsen *et al*. 2004). *NKX2-2* is a gene associated with gliogenesis and may play a role in differentiation of oligodendrocyte progenitor cells (Rousseau *et al*. 2006).

**Table 5:** Transcription factors enriched at days 21, 22 and 42 in chicken (*Gallus gallus*) pituitary glands.

**Day(s)**	**# of enriched genes**	**TF symbol**	**TF description**
Days 21, 22 and 42	25 genes	NKX2-2	NK2 homeobox 2
		MYT1	Myelin transcription factor 1
Days 21 and 22	295 genes	FOXC2	Forkhead box C2
		FOXL1	Forkhead box L1
		IRX1	Iroquois homebox 1
		LHX2	LIM homeobox 2
		MSX1	Msh homeobox 1
		NPAS3	Neuronal PAS domain protein 3
		NTN1	Netrin 1
		OLIG3	Oligodendrocyte transcription factor 3
		PHOX2B	Paired like homeobox 2B
		POU3F1	POU class 3 homeobox 1
		SOX8	SRY-box 8
		ST18	Suppression of tumorigenicity 18, zinc finger
		TBX22	T-box 22
		VAX1	Ventral anterior homeobox 1
Day 21	74 genes	NR2E1	Nuclear receptor subfamily 2 group E member 1
Day 22	92 genes	DLX1	Distal-Less homeobox 1
		HES6	Hes family BHLH transcription factor 6
		LOC427656	Forkhead box protein L1-like
		SOX10	SRY-box 10
Day 42	407 genes	CHURC1	Churchill domain containing 1
		DMBX1	Diencephalon/mesencephalon homeobox 1
		ESR1	Estrogen receptor 1
		FOS	FBJ murine osteosarcoma viral oncogene homolog
		FOXG1	Forkhead box G1
		FOXL2	Forkhead box L2
		HES5	Hes family BHLH transcription factor 5
		IRF6	Interferon regulatory factor 6
		INHBA	Inhibin beta A
		ISL1	ISL LIM homeobox 1
		LHX3	LIM homeobox 3
		NEUROD1	Neuronal differentiation 1
		NEUROG1	Neurogenin 1
		NHLH2	Nescient helix-loop-helix 2
		NR4A3	Nuclear receptor subfamily 4 group A member 3
		NR5A1	Nuclear receptor subfamily 5 group A member 1
		PAX6	Paired box 6
		PITX1	Paired like homeodomain 1
		PITX2	Paired like homeodomain 2
		POU1F1	POU class 1 homeobox 1
		PRRX2	Paired related homeobox 2
		RAX	Retina and anterior neural fold homeobox
		RBPJL	Recombination signal binding protein for immunoglobulin kappa J region like
		RFX6	Regulatory factor X6
		RHOXF1	Rhox homeobox family member 1
		SIX1	SIX homeobox 1
		SIX3	SIX homeobox 3
		SMAD7	SMAD family member 7
		TBX19	T-box 19
		TBX20	T-box 20

Transcription factors (TF) present within enriched genes separated by day. # of enriched genes corresponds to total genes (including TF) that were enriched in each category.

## Discussion

### Day 21 and 22: shared and unique enriched genes

Relative tissue expression analysis for days 21 and 22 results in similarly enriched genes involved in nervous tissue development (axon formation and neurite outgrowth) and signaling (GABA and glutamate receptors). Acid sensing ion channel subunit family member 4 (*ASIC4*) is a sodium channel involved in signal transduction and is known to have highest expression in the brain (Grunder *et al*. 2001). Histamine Receptor H3 (*HRH3*) is a receptor for histamine, which is involved in neurotransmitter release and pituitary hormone secretion (Nuutinen & Panula 2010). Uniquely enriched genes at days 21 and 22 indicate a shift from neuronal morphogenesis and synapse organization at day 21 to neurite outgrowth and myelination at day 22. This may be indicative of a developmental change from morphogenesis to maintenance of the nervous tissue, and it is unclear if this fluctuation between morphogenesis and maintenance continues throughout development. Two immune-related genes were also enriched in day 22: *HDAC11* and *CD59*. Histone deacetylases are a group of enzymes that interact with histones to regulate transcription. *HDAC11* is the most novel of the group and is placed in its own category, class IV. *HDAC11* regulates the expression of interleukin 10 (*IL10*), and immune tolerance and suppression can promote *IL10* expression in mice macrophages, whereas chromatin changes in macrophages increased *HDAC11* recruitment to *IL10* gene, inhibiting *IL10* production (Villagra *et al*. 2009, Wang *et al*. 2011). Studies investigating *HDAC11* found abundant transcripts are detected in the brain, skeletal muscle, kidney and testis; however, little is known about its function in healthy tissues (Deubzer *et al*. 2012). Because *HDAC11* is a unique histone deacetylase, and it is possible it has functions that are unknown or not typically associated with histone deacetylases (Gao *et al*. 2002). *CD59* is a cell surface glycoprotein that inhibits the assembly of the membrane attack complex (MAC) during complement attack (Huang *et al*. 2001). Activation of the complement attack can lead to inflammation and lysis of cells and needs to be highly regulated. *CD59* inhibits MAC osmolytic pore formation thereby protecting cells (Liu *et al*. 2007). It is possible that development of the pars tuberalis and the macrophages within this region result in enrichment of these immune-related genes at days 21 and 22 but not day 42 and may provide anti-inflammatory functions during neuronal development. Overall, the focus of the pituitary gland at days 21 and 22 appears to be focused on developing and maintaining nervous tissue connections and signaling.

### Day 42 enriched genes

By day 42, gene expression suggests a shift in the focus of the pituitary gland through maturation from strictly nervous tissue development and signaling to endocrine- and hormone-related functions. Most notably, there is the robust increase in expression of peptide hormone genes *GH*, *PRL*, *TSHB* and *FSHB*. This change in enriched genes could be in preparation for reproduction. In addition to receptors for releasing hormones from the hypothalamus, and beta subunits of follicle-stimulating hormone (*FSH*) and thyroid-stimulating hormone (*TSH*), two other genes of interest are carboxypeptidase E (*CPE*) and deiodinase type 2 (*DIO2*). *CPE* is a carboxypeptidase B-like enzyme involved in peptide and neurotransmitter synthesis and processing of neuropeptides. Immunohistochemical studies in the rat have shown immunoreactivity within all components of the hypothalamic-neurohypophyseal system (Lynch *et al*. 1990). *CPE* has prohormone processing functions in neuroendocrine cells and directs proopiomelanocortin (*POMC*) to the secretory pathway where it is later cleaved to produce ACTH (Dhanvantari *et al*. 2002). *DIO2* converts thyroxine (T_4_) to triiodothyronine (T_3_), the main metabolic hormone important for neuronal development and differentiation in the brain (Noda 2015). Circulating levels of T_4_ are transported across the blood–brain barrier to astrocytes by organic anion-transporting polypeptide (OATPs) transporters and converted by *DIO2* to T_3_. T_3_ is important for neuron, microglia, astrocyte and oligodendrocyte development and function and thyroid hormone dysfunction can influence these processes (Karlsson *et al*. 2016). *DIO2* expression is also involved in seasonal reproduction in birds and is expressed during long day conditions. The increased day length leads to an increase in T_3_ which then stimulates the release of FSH and luteinizing hormone (LH) for reproduction (Karlsson *et al*. 2016). The exact location of *DIO2* expression in the brain is still under investigation although it is thought to be exclusively expressed in the glial compartment (Egri *et al*. 2016). This research shows expression in the pituitary gland suggesting future work should identify the location of *DIO2* mRNA within the pituitary.

Dopamine receptor D2 (*DRD2*) and somatostatin receptor 5 (*SSTR5*) are receptors found in the brain. *DRD2* is expressed in lactotrophs and inhibits prolactin release. *SSTR5* is involved in the regulation of GH and TSH. Both *DRD2* and *SSTR5* are also involved in ACTH release in pituitary tumors (Tani *et al*. 2011).

Calcium-dependent secretion activator (*CADPS*) and synaptotagmin IV (*SYT4*) gene products are involved in the calcium-dependent vesicle-mediated exocytosis of neurotransmitters and peptides in conjunction with plasma membrane-associated and vesicle integral membrane SNAREs. *CADPS* is essential in exocytosis of dense-core vesicles (used for exocytosis of biogenic amines and peptides) in the pituitary gland and is involved in secretion of serotonin and other neurotransmitters in *Caenorhabditis elegans* and *Drosophila melanogaster* (Wassenberg & Martin 2002)*. SYT4* is a synaptotagmin that is a member of the vesicle integral membrane SNARE genes. Once hormones are produced, they are stored in secretory granules where extracellular signals induce fusion with the plasma membrane and subsequent release (Moore *et al*. 2002). *SYT4* is an inhibitor of regulated exocytosis (Wassenberg & Martin 2002).

Cocaine- and amphetamine-regulated transcript (*CARTPT*) is an anorectic peptide that is expressed in the hypothalamus and the anterior pituitary. In the anterior pituitary, *CARTPT* is present in corticotrophs, gonadotrophs, lactotrophs and thyrotrophs. *CARTPT* mRNA expression is regulated by corticosterone and corticotropin-releasing hormone (CRH) from the hypothalamus via corticotropin-releasing hormone receptor 1 (CRHR1). Intracerebroventricular injections of *CARTPT* lead to elevated ACTH and corticosterone levels, suggesting that *CARTPT* can activate the hypothalamic–pituitary–adrenal (HPA) axis and may be involved in the processes of the HPA axis, such as the stress response (Mo *et al*. 2015). Similarly, gastrin-releasing peptide (*GRP*) is a neuropeptide that affects AVP and CRH secretion from the hypothalamus and acts directly at the pituitary gland by increasing the effects of AVP and CRH release on corticotrophs (Olsen *et al*. 1992).

### Day 21, 22 and 42: 25 shared enriched genes

Of the 25 shared genes between days 21, 22 and 42, genes related to the extracellular matrix (ECM) were enriched. The ECM organizes and fills the space between cells and is composed of glycosaminoglycans and fibrous proteins such as collagen and elastin. The ECM not only provides support to surrounding cells but also influences proliferation, differentiation and survival (Alberts *et al*. 2002). ECM gene collagen type XI alpha 1 (*COL11A1*) encodes for an alpha chain of a minor fibrillary collagen and is important for extracellular matrix organization and has been associated with cell proliferation and migration (Mio *et al*. 2007, Shen *et al*. 2016). Matrilin-4 (*MATN4*) is in the matrilin family of proteins and is a recently characterized extracellular matrix protein that is expressed in a variety of tissues, including the brain (Wagener *et al*. 2001). The pituitary gland is involved in nervous tissue and endocrine signaling and displays tissue plasticity in response to signals from the hypothalamus. This plasticity is necessary to accommodate the conditions of development (i.e. initiation reproductive maturation), and the ECM is an important component of tissue growth and maintenance throughout life. In addition to enrichment for specific ECM genes, there are also enriched genes involved in cell proliferation and differentiation. Glypican 3 (*GPC3*) interacts with the ECM to influence cell cycle control. *GPC3* is a glypican member within the family of heparin sulfate proteoglycans anchored to the cell surface that interact with extracellular ligands to influence cell processes, like growth, and function during morphogenesis and tissue repair (Bernfield *et al*. 1999). Glypicans are predominately expressed in the central nervous system and have been associated with negative regulation of cell proliferation (Sung *et al*. 2003).

### Enriched transcription factor genes

Transcription factors (TF) control the rate of transcription and are essential to pituitary cell development, differentiation and function (Mizokami *et al*. 2008). Because of their influential functions, transcription factors that may be associated with particular points in development of the pituitary gland were identified. Within the 295 enriched genes shared between days 21 and 22, 14 transcription factors were enriched. Netrin 1 laminin related (*NTN1*) and ventral anterior homeobox 1 (*VAX1*) are both related to axon guidance in the developing nervous system. *NTN1* specifically directs the development and migration of luteinizing hormone-releasing hormone neurons in the forebrain (Schwarting *et al*. 2004). During embryonic development, *VAX1* is responsible for limiting the areas in which fibroblast growth factor 10 (*FGF10*) can induce pituitary-specific development and ensures that only a single pituitary develops (Bharti *et al*. 2011). Paired like homeobox 2B (*PHOX2B*) is critical during embryonic development to regulate neural tube progenitor cells to exit the cell cycle and differentiation to a particular type of neuron (Dubreuil *et al*. 2000). Two transcription factors are associated with neurogenesis (*POU3F1* and *NPAS3*), whereas suppression of tumorigenicity 18 (*ST18*) is uniformly expressed in the brain and is associated with pro-apoptotic and pro-inflammatory functions in fibroblasts (Yang *et al*. 2008). Lastly, two genes are associated with the alpha peptide of glycoprotein hormones (CGA). In humans and mice, Msh Homeobox 1 (*MSX1*) has been associated with CGA expression and is localized in growth hormone and thyroid-stimulating hormone cells of the anterior pituitary (Mizokami *et al*. 2008). LIM Homeobox 2 (*LHX2*) is a transcription factor known to repress the CGA promoter (Susa *et al*. 2006) and has a direct role in the development of the posterior pituitary gland (Davis *et al*. 2010). Several transcription factors normally associated with embryonic development at days 21 and 22 were identified; however, the presence of these genes in post-hatch birds suggests these genes may continue to influence development through axonal guidance (*VAX1*) and limiting the growth of pituitary-related cell types (*NTN1*).

Thirty transcription factors are enriched at day 42 and several are involved in endocrine-related processes. POU class 1 homeobox 1 (*POU1F1*) is a pituitary-specific transcription factor that is responsible for the activation of *GH*, *PRL* and *TSHB* gene transcription (Pfaffle *et al*. 1993) and is critical for proper pituitary development (Zhu *et al*. 2005). Nuclear receptor subfamily 5 group A member 1 (*NR5A1*) functions in the development of LH- and FSH-expressing cells and T-box 19 (*TBX19*) is restricted to corticotrophs where it binds the POMC promoter and affects the expression of ACTH (Zhu *et al*. 2005). Lim homeobox 3 (*LHX3*) and Forkhead box L2 (*FOXL2*) are associated with positive regulation of the CGA promoter (Susa *et al*. 2006). Neuron differentiation 1 (*NEUROD1*) has been identified as a transcription factor involved in corticotroph differentiation and POMC gene expression (Tani *et al*. 2011). Regulatory factor X6 (*RFX6*) is a newly characterized transcription factor expressed primarily in pancreatic tissues and may regulate pancreatic development. The paralogous gene, regulatory factor X7 (*RFX7*), is also recently characterized in humans and is expressed in several tissues, particularly the brain (Aftab *et al*. 2008) but was not shown to be enriched in our data. It is surprising that a transcription factor most commonly associated with the pancreas (*RFX6*) is present in the pituitary gland at day 42 rather than the paralogous transcription factor normally associated with brain tissue (*RFX7*); however, the brain is an insulin-sensitive organ and perhaps *RFX6* is present in the chicken pituitary gland to assist in insulin-related processes (i.e. insulin receptors, glucose uptake).

Two pituitary enriched transcription factors found in all days, *MYT1* and *NXK2-2*, are both related to oligodendrocyte differentiation, proliferation and maturation. *MYT1* is a DNA-binding protein with an expression pattern that indicates a potential role in regulating oligodendrocyte differentiation. In the developing and adult central nervous system, *MYT1* expression correlates with oligodendrocyte cell growth (Nielsen *et al*. 2004). *NKX2*-*2* is associated with gliogenesis and may play a role in the differentiation of oligodendrocyte progenitor cells (Rousseau *et al*. 2006). The presence of these TFs in all days indicates the need for continued oligodendrocyte proliferation. This proliferation may accommodate new connections between the pituitary gland and other brain regions, specifically the hypothalamus, and may be important for establishing and maintaining the connections within the hypothalamo–pituitary axis. Other neuronal-related TFs independent of those identified at days 21 and 22 were enriched at day 42 (i.e. *CHURC1*, *DMBX1*). These transcription factors should be explored further to determine the temporal relationship as it is possible these TFs are necessary to establish the endocrine-related processes within the pituitary gland. TF results also point to a maturation shift from prominently neuronal-related TFs at day 21/22 to endocrine-related TFs at day 42.

In summary, there is a transcriptome focus on nervous tissue development, signaling and maturation at days 21 and 22 post-hatch. By day 42, there is a shift from strictly nervous tissue gene expression toward endocrine-related processes, possibly indicating maturation in preparation for reproduction. The results of this study lead to valuable insight into the genes involved in the pituitary gland at several points in development, particularly related to maturation. Future work aims to utilize fluorescent in situ hybridization techniques to investigate the location of particular mRNA transcripts within the pituitary such as *DIO2* and *RFX6*. Future work should investigate the transcriptomic differences, particularly related to neuronal and endocrine maturation, as a function of age in male broiler chicken pituitary glands by collecting additional time points in future trials. The pituitary gland is a tissue that contains many different cell types and exerts many physiological changes based on the input received from the body and the environment. Whole tissue transcriptomics is informative but cellular transcriptomic changes that may have important physiological impacts may be lost or not as robust. Single-cell transcriptomic data could further elucidate the cellular transcriptomic differences that may influence the development and function of the pituitary gland. Single-cell transcriptome analysis would allow for identification of more prevalent cell types and the transcriptomic changes throughout development.

## Supplementary data

This is linked to the online version of the paper at http://dx.doi.org/10.1530/JME-16-0186.

## Declaration of interest

The authors declare that there is no conflict of interest that could be perceived as prejudicing the impartiality of the research reported.

## Funding

This project was supported by Agriculture and Food Research Initiative Competitive Grant 2011-67003-30228 from the United States Department of Agriculture National Institute of Food and Agriculture.

## Author contribution statement

E M P, C J S and S J L conceived and designed the experiments. E M P and C J S performed the experiments. E M P and C J S analyzed the data. E M P, C J S and S J L contributed reagents/materials/analysis tools. E M P wrote the paper.
